# Targeting PD‐L1 for Ischemic Stroke Recovery: Age‐Dependent Modulation of Immune and BBB Pathways

**DOI:** 10.1111/cns.70523

**Published:** 2025-07-23

**Authors:** Hang Hang, Can Xu, Likun Wang, Cuiying Liu, Rongping Zhang, Guofeng Wu, Heng Zhao

**Affiliations:** ^1^ Emergency Department the Affiliated Hospital of Guizhou Medical University Guiyang Guizhou China; ^2^ Beijing Institute of Brain Disorders, Laboratory of Brain Disorders, Ministry of Science and Technology, Joint Innovation Center for Brain Disorders Capital Medical University Beijing China; ^3^ College of Chinese Materia Medica and Yunnan Key Laboratory of Southern Medicine Utilization, Yunnan University of Chinese Medicine Kunming China; ^4^ School of Nursing, Capital Medical University Beijing China; ^5^ Department of Neurology and Neurosurgery Tongren People's Hospital Tongren Guizhou China

**Keywords:** age‐dependent immune response, blood–brain barrier integrity, ischemic stroke, neuroinflammation, PD‐L1 monoclonal antibody

## Abstract

**Objective:**

Aging has a profound impact on the pathophysiology of ischemic stroke and the effectiveness of therapeutic interventions. This study aims to evaluate the therapeutic efficacy of programmed death‐ligand 1 (PD‐L1) monoclonal antibody (mAb) in modulating immune responses and neurovascular repair following ischemic stroke, with a focus on age‐dependent differences.

**Methods:**

Young and aged mice were subjected to middle cerebral artery occlusion (MCAO) followed by PD‐L1 mAb treatment. RNA sequencing, immunofluorescence, and molecular analyses were employed to assess immune modulation, blood–brain barrier (BBB) integrity, and functional recovery.

**Results:**

RNA sequencing revealed significant differential gene expression in ischemic brain tissues, with CD274 (PD‐L1) prominently upregulated among immune checkpoint‐related genes in young mice. Immunofluorescence confirmed PD‐L1 expression in microglia/macrophages, with significantly higher upregulation in young mice. PD‐L1 mAb treatment showed superior efficacy in young mice, significantly reducing infarct volume, enhancing neurological recovery, and preserving BBB integrity through greater upregulation of tight junction proteins such as ZO‐1, Claudin‐5, and Occludin compared to aged mice. It also more effectively reduced neuroinflammation, apoptosis, and pro‐inflammatory cytokines (TNF‐α, IL‐1β), eliciting stronger spleen responses in young mice. These findings underscore the age‐dependent advantages of PD‐L1‐targeted therapies for ischemic stroke recovery.

**Conclusions:**

PD‐L1 plays a critical role in ischemic stroke recovery, with PD‐L1 mAb treatment demonstrating age‐dependent therapeutic efficacy by enhancing BBB integrity, reducing neuroinflammation and apoptosis, and modulating peripheral immune responses.

## Graphical abstract

PD‐L1 plays a critical role in ischemic stroke recovery, with PD‐L1 mAb treatment demonstrating age‐dependent therapeutic efficacy by enhancing blood–brain barrier integrity, reducing neuroinflammation and apoptosis, and modulating peripheral immune responses.

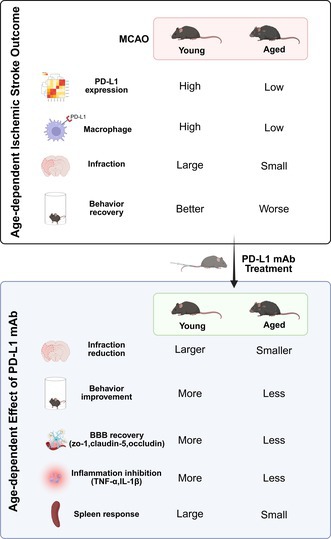

## 1 Introduction

Ischemic stroke remains a significant global health challenge due to its complex pathophysiology and limited therapeutic options [1]. Poststroke immune activation is a double‐edged sword: while it contributes to secondary neuronal damage, it also plays a critical role in initiating tissue repair [2, 3]. Effectively modulating immune responses to reduce inflammation and enhance recovery has been a key focus of research. Programmed death‐ligand 1 (PD‐L1) has emerged as a crucial regulator of immune suppression, primarily through its interaction with programmed death‐1 (PD‐1) limits excessive T cell activation and mitigates secondary neuronal injury [4]. Despite limited studies on PD‐L1 in ischemic stroke [5], the potential of PD‐L1 as an immunomodulatory therapeutic target for ischemic stroke remains well explored.

Age is a major determinant of ischemic stroke outcomes and significantly influences the efficacy of therapeutic interventions [6]. Aging is associated with profound physiological changes, including immune dysregulation, reduced neuroplasticity, and vascular dysfunction, all of which exacerbate the severity of ischeimc stroke and impair recovery [7, 8]. Research has shown that aged individuals experience heightened neuroinflammation following ischemic stroke, accompanied by diminished anti‐inflammatory responses and impaired repair mechanisms [9]. Additionally, the aging blood–brain barrier (BBB) is more susceptible to damage, leading to increased permeability, inflammatory infiltration, and cerebral edema, further impeding recovery [10].

While aging is a well‐established determinant of ischemic stroke outcomes, its impact on infarct volume remains inconclusive. Some studies have reported that aged mice develop larger infarcts than younger counterparts, likely due to exacerbated vascular dysfunction and less complete reperfusion owing to inherent limitations [11, 12]. Conversely, other studies have observed a paradoxical reduction in infarct size in aged animals despite more severe neurological deficits—a finding attributed to age‐related differences in neurovascular responses, inflammatory dynamics, and tissue resilience [13, 14, 15, 16]. These discrepancies highlight the complexity of ischemic stroke pathophysiology in aging populations and underscore the need for a more precise characterization of age‐dependent mechanisms governing infarct evolution and functional impairment. These differences emphasize the importance of understanding how aging affects PD‐L1 mAb efficacy.

This study systematically investigates the therapeutic effects of PD‐L1 mAb in ischemic stroke, focusing on the age‐dependent differences in immune modulation, BBB integrity, and functional recovery. By directly comparing young and aged mice, this study aims to elucidate how aging alters the mechanisms and efficacy of PD‐L1‐mediated neuroprotection. These findings not only expand our understanding of PD‐L1's therapeutic potential but also provide critical insights for developing age‐specific treatment strategies for ischemic stroke.

## 2 Materials and Methods

### 2.1 Animals

Experiments were performed using young (8–10 weeks old) and aged (18 months old) male C57BL/6J mice, which were purchased from Vital River Laboratory (Beijing, China). All mice were housed in standard plastic cages (three or four per cage) and maintained under a 12‐h light/dark cycle starting at 6:00 a.m. The mice had free access to food and water, and the temperature and humidity were maintained according to the standards set by the laboratory for experimental animals. All experiments were conducted following the National Institutes of Health Guide for the Care and Use of Laboratory Animals in China and in accordance with the ARRIVE (Animal Research Reporting In Vivo Experiments) guidelines approved by the Capital Medical University Animal Ethics Committee (NO. AEEI‐2023‐229). All efforts were made to minimize the number of animals used and their suffering.

### 2.2 Transient Middle Cerebral Artery Occlusion

Ischemic stroke was induced with transient middle cerebral artery occlusion (tMCAO) as previously described [17]. Briefly, mice were anesthetized using isoflurane 5% for induction and 2% for maintenance, respectively; the core body temperature was maintained at 37°C ± 0.5°C during the surgical procedures. A ventral midline neck incision was made under the operating microscope to expose and isolate the left common carotid artery (CCA) and external carotid artery (ECA). The ECA and the distal portion of the CCA were ligated. A loose tie was placed around the CCA proximal to its bifurcation and the internal carotid artery (ICA). Silicone‐coated nylon monofilament (0.21‐ and 0.23‐mm tip diameter, RWD, China) was inserted into the ICA incision in young and aged mice, respectively. Once the filament was in place, the tie around the CCA was tightened, and the tie around the ICA was removed. The filament was then gently advanced up the ICA, approximately 9–10 mm, until resistance was encountered. Successful occlusion was verified by a laser speckle imaging system (RFLSI‐ZW, RWD, China). The mice that exhibited less than 80% CBF reduction were excluded. The ratio of mice with successful reperfusion after stroke was more than 90%. After 45 min of induced ischemia, the filament was removed. Sham‐operated mice underwent the same procedure, but without monofilament insertion. Mice were euthanized with isoflurane after 24‐ and 72‐h reperfusion.

### 2.3 PD‐L1 mAb Administration

Mice from both age groups were randomly allocated into four experimental groups: Sham, MCAO, MCAO treated with vehicle, and MCAO treated with monoclonal anti‐PD‐L1 antibody (PD‐L1 mAb). The PD‐L1 mAb group received 50 μg of PD‐L1 mAb (BP0101, clone 10F.9G2, BioXcell, NH, USA). In comparison, the vehicle group received 50 μg of isotype‐matched control (anti‐Keyhole Limpet Hemocyanin [KLH], clone LTF‐2, BioXcell, NH, USA), both in a total volume of 100 μL by tail intravenous injection, administered 1 and 24 h after reperfusion. The MCAO group underwent a tMCAO procedure, while the sham group underwent a sham operation.

### 2.4 Neurological Function Assessment

All assessments were performed by investigators who were blinded to the treatment groups. On Days 1, 2, and 3 poststroke, the cylinder test, foot‐fault test, and modified neurological severity score (mNSS) were conducted to evaluate motor function and neurological impairment [18].

### 2.5 Laser Speckle Contrast Imaging (LSCI) for Blood Flow Monitoring

LSCI was used to assess blood flow dynamics following ischemic stroke. Mice were anesthetized, and the skull was exposed for imaging using a high‐resolution LSCI system with LSCI‐V 1.0.0 software (RWD Life Science, China). CBF was measured using LSCI prior to MCAO, 10 min after occlusion, and 20 min after reperfusion in each group. Blood flow was quantified based on standard protocols [19].

### 2.6 Assessment of Infarct Volume

Brain infarction at 3 days poststroke was evaluated using 2% (w/v) 2,3,5‐triphenyl tetrazolium chloride (TTC) staining (Sigma‐Aldrich, MO, USA) as previously described [20]. Following fixation in 4% paraformaldehyde, TTC‐stained brain slices were scanned using a Pannoramic MIDI imaging system. Infarct volume was quantified using ImageJ software (NIH, USA).

### 2.7 RNA Sequencing

On Day 1 poststroke, fresh peri‐infarct brain tissue was collected for sequencing analysis. RNA from the cortical peri‐infarct tissue of 10 mice was extracted using Trizol (Invitrogen, USA). RNA quality was assessed using the Agilent 2100 Bioanalyzer. Poly(A)‐selected RNA‐seq libraries were prepared with the Fast RNA‐seq Lib Prep Kit V2 and sequenced on an Illumina NovaSeq platform (150‐bp paired‐end reads). Reads were filtered using fastp (v0.19.7), aligned to the *Mus musculus* genome (GRCm38) with HISAT2 (v2.0.5), and quantified using featureCounts (v1.5.0‐p3). Differentially expressed genes (DEGs) were identified using DESeq2 (v1.20.0).

### 2.8 Assessment of Blood–Brain Barrier (BBB) Permeability by Evans Blue Dye Extravasation

BBB permeability was assessed by measuring Evans blue (EB) dye extravasation [20]. Mice received an intravenous injection of 2% EB dye (Sigma‐Aldrich, MO, USA) at 4 mL/kg, 3 days after MCAO. One hour later, the mice were perfused with cold 0.9% NaCl and then sacrificed. The brain hemispheres were collected, weighed, and homogenized in 2 mL of N, N‐dimethylformamide (Sigma‐Aldrich, USA). The homogenates were incubated at 60°C for 72 h. After centrifugation (5000 rpm, 10 min, 4°C), the absorbance of the supernatant was measured at 620 nm using a spectrophotometer. The EB content was quantified by comparing the absorbance to a standard curve, and the results were expressed as micrograms of EB per gram of brain tissue.

### 2.9 Western Blot Analysis

As previously described [20], protein lysates from ischemic hemisphere samples were prepared using RIPA buffer (Sigma‐Aldrich, MO, USA) with protease and phosphatase inhibitors. Protein concentration was measured using the BCA assay (Bio‐Rad, CA, USA). Equal amounts of protein were separated by SDS‐PAGE, transferred to PVDF or nitrocellulose membranes, and blocked with 5% nonfat milk in TBST for 1 h. Membranes were incubated overnight at 4°C with primary antibodies: ZO‐1, Claudin‐5, Occludin (1:1000, Cell Signaling Technology, USA) and β‐actin (1:5000, Proteintech, China). After washing, membranes were incubated with HRP‐conjugated secondary antibodies for 2 h at room temperature. Protein bands were visualized using ECL reagent (ZETA‐life, USA). Band intensities were quantified with ImageJ software (NIH, USA) and normalized to β‐actin.

### 2.10 Immunofluorescence Staining

Mice were perfused with 4% paraformaldehyde via cardiac perfusion, fixed, and embedded in paraffin for sectioning (4 μm thickness), followed by permeabilization and blocking in PBS containing 1% Triton X‐100 and 10% normal donkey serum for 30 min at room temperature. The samples were then incubated overnight at 4°C with primary antibodies: Iba‐1 (1:800, Proteintech, China), iNOS (1:500, Abcam, USA), PD‐L1 (1:400, Invitrogen, USA), CD31 (1:800, Abcam, USA), ZO‐1 (1:500, Abcam, USA), Claudin‐5 (1:200, Abcam, USA). After washing with PBS containing 0.3% Triton X‐100, the sections were incubated with the appropriate fluorescence‐conjugated secondary antibodies (1:400, Proteintech, China) for 1 h at room temperature in the dark. DAPI (1:1000, Sigma‐Aldrich, USA) was used to stain nuclei. Fluorescence images were captured using a fluorescence microscope (Olympus IX73). Immunopositive cells were quantified by a blinded investigator using NIH ImageJ software. Three randomly selected fields from the cortical infarct core and border zone of each section were analyzed.

### 2.11 Cytokine Enzyme‐Linked Immunosorbent Assay

Blood plasma was collected. Concentrations of TNF‐α and IL‐1β were measured with commercial ELISA quantification kits (MEIMIAN Bio, China) according to the manufacturer's instructions.

### 2.12 Bioinformatics Analysis

DEGs were filtered with adjusted *p* value < 0.05 and |log2 fold change| ≥ 0.263. Volcano plots were generated using ggplot2 (v3.3.2). Gene Ontology (GO) and Kyoto Encyclopedia of Genes and Genomes (KEGG) enrichment analyses were performed using the clusterProfiler package (v3.14.3). Immune checkpoint‐related genes and tight junction‐related genes were downloaded from previous studies and Gene Cards [21]. Protein–Protein Interaction (PPI) networks were constructed using the string database and visualized with Cytoscape (v3.9.1).

### 2.13 Statistical Analysis

All data were analyzed using SPSS 23.0 or GraphPad Prism 9.5 (San Diego, CA, USA) and are presented as mean ± SD. Data were tested for normality using the Shapiro–Wilk test prior to parametric analysis. For normally distributed data, two groups were compared using Student's *t*‐test, while one‐way ANOVA with Bonferroni post hoc test was used for multiple groups. Two‐way ANOVA was applied to evaluate interactions between factors where appropriate. Kaplan–Meier survival curves were analyzed using the log‐rank (Mantel–Cox) test. A two‐tailed *p* < 0.05 was considered statistically significant.

## 3 Results

### 3.1 Transcriptomic Profiling Reveals Immune Checkpoint Upregulation in Young Mice After Ischemic Stroke

**FIGURE 1:**
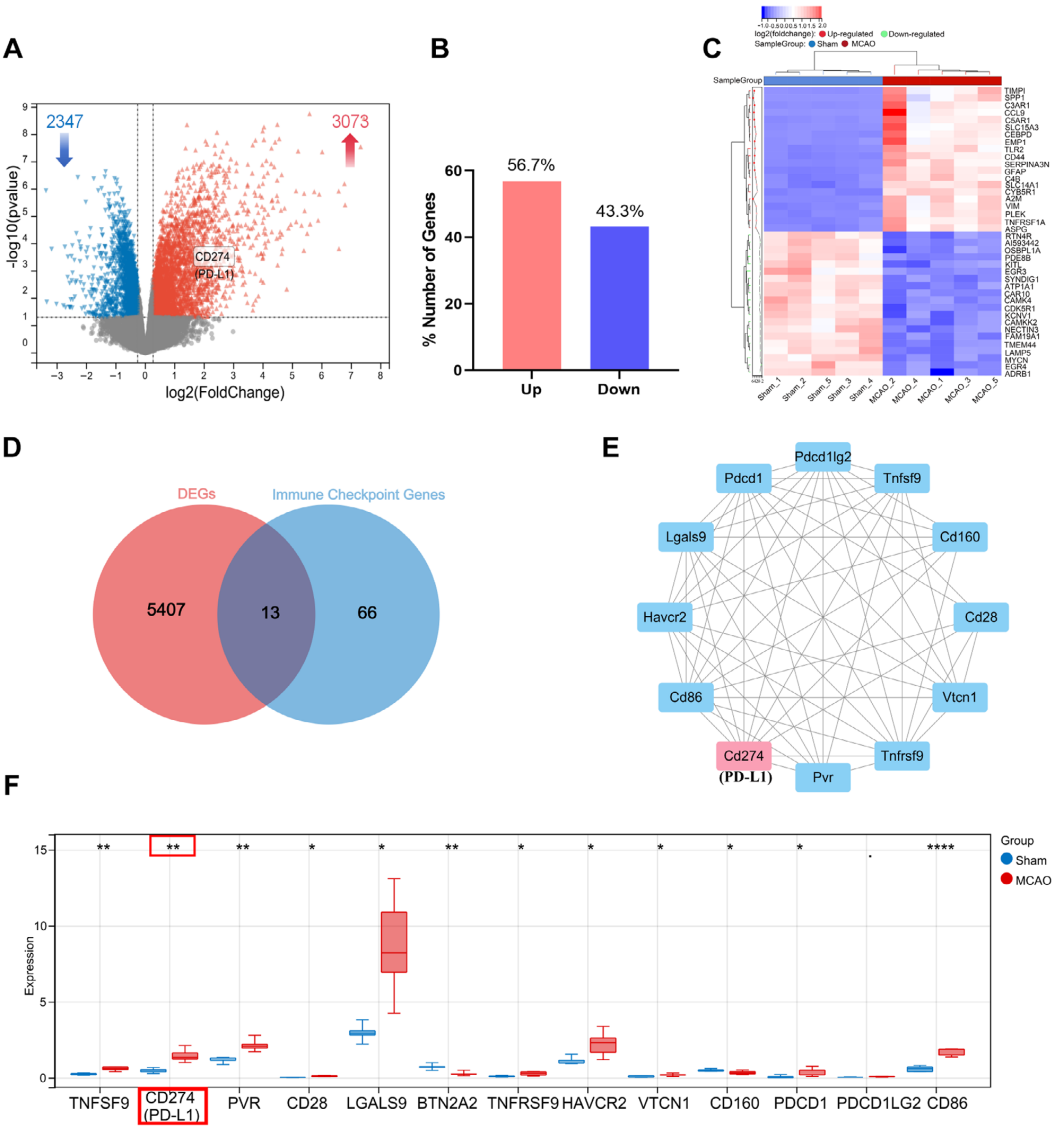
Transcriptomic analysis reveals significant changes in immune checkpoints in ischemic brain tissues of young mice. (A) Volcano plot highlights CD274 (PD‐L1) among the upregulated genes, showing the distribution of 3073 upregulated and 2347 downregulated DEGs (|FC| > 1.2, *p* < 0.05). (B) Bar chart depicts the proportional distribution of DEGs, with 56.7% upregulated and 43.3% downregulated. (C) Heatmap illustrates the expression of the top 20 DEGs between the MCAO and sham groups (*n* = 5). (D) Venn diagram identifies 13 immune checkpoint‐related genes by intersecting DEGs with an immune checkpoint gene set. (E) PPI network highlights the relationships among these 13 genes, with color intensity reflecting their centrality. (F) Box plot shows significant upregulation of CD274 (PD‐L1) in the MCAO group compared to the sham group (*p < 0.05, **p < 0.01, ***p < 0.001).

We aimed to explore whether PD‐L1 is involved in ischemic stroke‐induced brain injury. Thus, we performed RNA sequencing using ischemic brain tissues of young animals. We identified significant differential gene expression in ischemic brain tissues compared to sham controls. We identified 3073 upregulated (56.7%) and 2347 downregulated (43.3%) genes (|FC| > 1.2, *p* < 0.05), with CD274 (PD‐L1) highlighted as an upregulated gene (Figure 1A,B). A heatmap of the top 20 differentially expressed genes (DEGs) demonstrated distinct clustering between the MCAO and sham groups (Figure 1C).

To investigate immune checkpoint involvement related to PD‐L1, DEGs were intersected with an immune checkpoint gene set, identifying 13 immune checkpoint‐related genes (Figure 1D). A protein–protein interaction (PPI) network revealed the central roles of these genes, with CD274 (PD‐L1) showing a prominent position based on network centrality (Figure 1E). Furthermore, PD‐L1 expression was significantly upregulated in the MCAO group compared to the sham group, as shown by box plot analysis (Figure 1F). These findings highlight the critical involvement of immune checkpoints, particularly PD‐L1, in ischemic stroke pathophysiology.

### 3.2 Ischemic Stroke Induces PD‐L1 Upregulation in Both Young and Aged Mice

**FIGURE 2:**
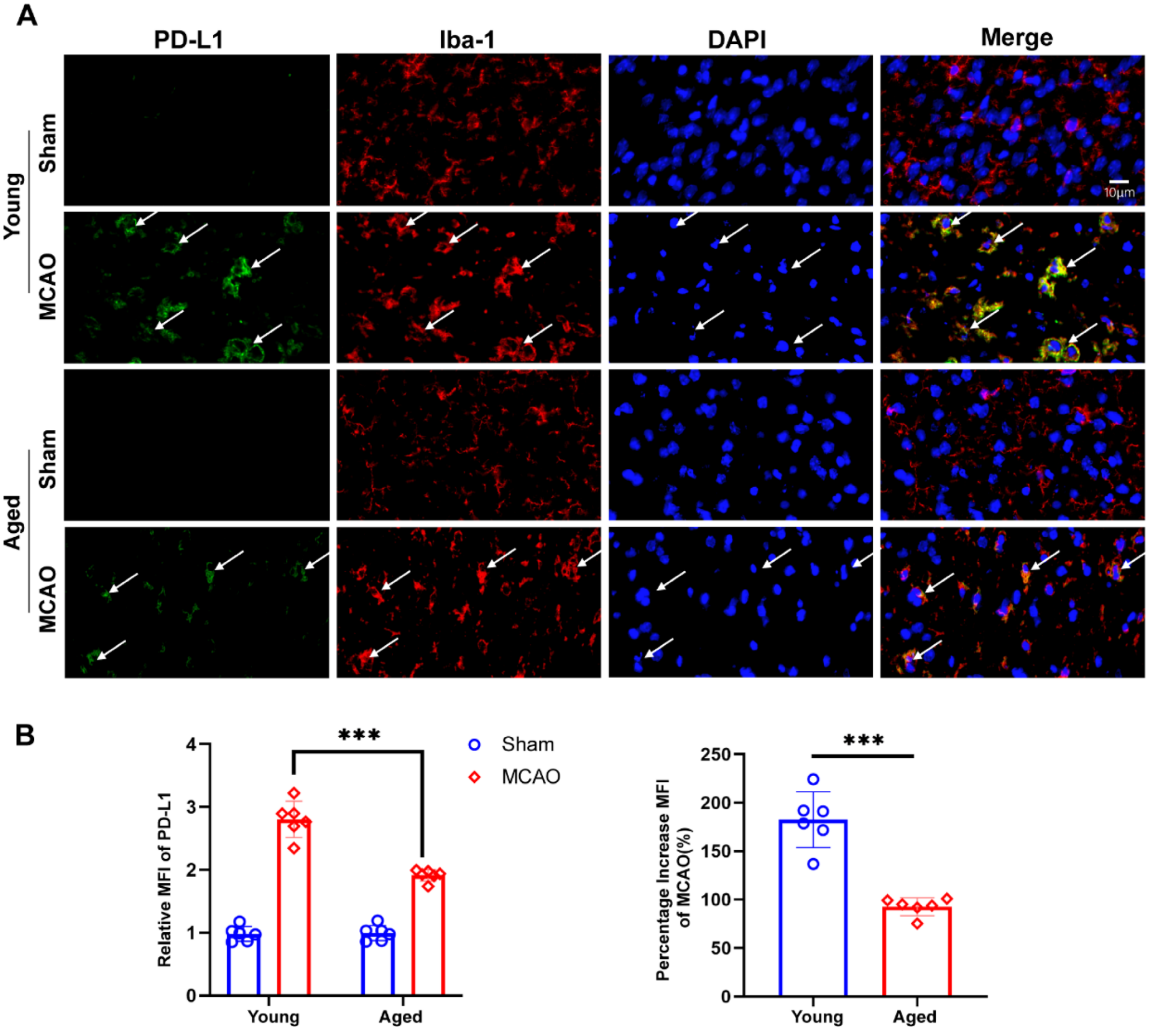
PD‐L1 expression is upregulated in the peri‐infarct brain regions of both young and aged mice following ischemic stroke. (A) Immunofluorescence staining of PD‐L1 (green) and Iba‐1 (red) in the peri‐infarct area of Sham and MCAO groups in young and aged mice; DAPI (blue) stains nuclei; arrows indicate PD‐L1‐positive cells. Scale bar = 10 μm. (B) Quantification of relative mean fluorescence intensity (MFI) of PD‐L1 shows a significant increase in MCAO groups, with a greater percentage increase in young mice compared to aged mice (*n* = 6, ***p < 0.001).

We performed immunofluorescence staining to investigate PD‐L1 expression after ischemic stroke in young and aged mice; we performed immunofluorescence staining to identify how ischemic stroke affects its expression. The results showed a significant upregulation of PD‐L1 expression in the peri‐infarct area of MCAO groups compared to sham groups in both young and aged mice (Figure 2A). PD‐L1 (green) colocalized with Iba‐1 (red), also a marker of microglia/macrophages, as indicated by arrows in the merged images, suggesting its expression in microglia/macrophages. Quantitative analysis revealed a significant increase in the relative mean fluorescence intensity (MFI) of PD‐L1 in the MCAO groups, with young mice exhibiting a substantially greater percentage increase than aged mice (Figure 2B). These findings indicate that ischemia‐induced PD‐L1 upregulation is more pronounced in younger mice, suggesting age‐dependent differences in PD‐L1 expression in response to ischemic stroke.

### 3.3 PD‐L1 mAb Treatment Improves Ischemic Stroke Outcomes More Effectively in Young Mice Than in Aged Mice

**FIGURE 3:**
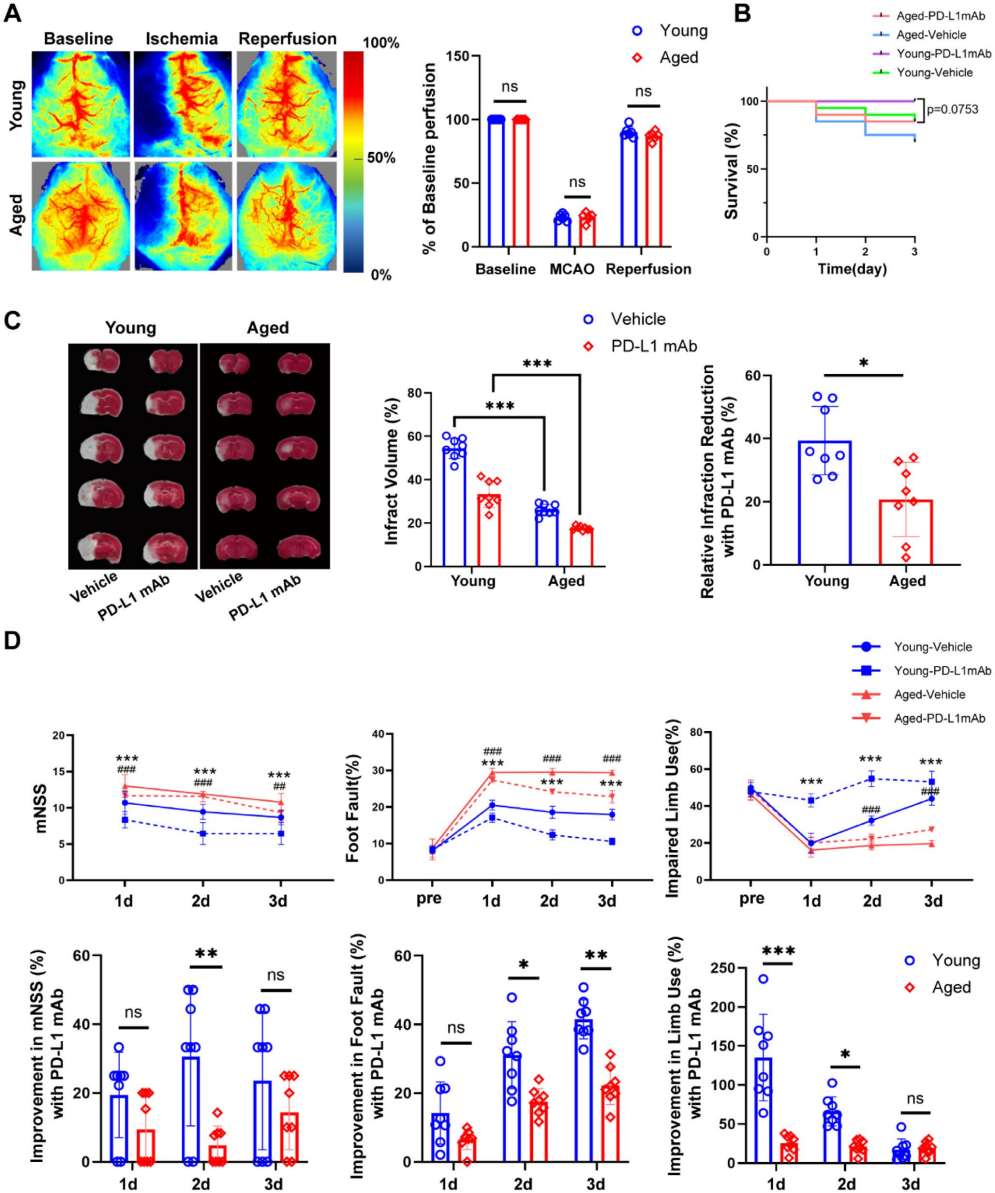
Age‐dependent effects of PD‐L1 mAb on ischemic stroke outcomes and cerebral recovery. (A) Laser speckle imaging shows baseline, ischemia, and reperfusion cerebral blood flow, with quantification indicating no significant differences between young and aged mice (*n* = 6). The redder the color, the higher the blood flow; the bluer the color, the lower the blood flow. (B) Kaplan–Meier survival analysis shows a trend toward improved survival in PD‐L1 mAb‐treated young mice compared to aged mice (*p* = 0.0753). (C) TTC staining reveals reduced infarct volume in PD‐L1 mAb‐treated young mice compared to vehicle controls and aged mice, with a significantly greater relative infarction reduction in young mice (*n* = 6). (D) Neurological assessments (mNSS, foot‐fault, and cylinder tests) demonstrate improved recovery in PD‐L1 mAb‐treated young mice compared to aged mice across 1–3 days following ischemic stroke (*n* = 8). *p < 0.05, **p < 0.01, ***p < 0.001; ns, not significant. In (D): * denotes comparisons between PD‐L1 mAb‐treated young and aged stroke mice; # denotes comparisons between vehicle‐treated young and aged stroke mice.

To further study the actual role of PD‐L1 in ischemic brain injury in young and aged mice, we used PD‐L1 mAb to investigate how it affects ischemic stroke outcomes. One week before ischemic stroke induction, mice underwent behavioral training, and baseline cerebral blood flow (CBF) levels were measured using laser speckle imaging (Figure 3A). PD‐L1 mAb or isotype control treatments were administered 1 h and 24 h following ischemic stroke, and neurological function and behavioral assessments were recorded over 3 days of reperfusion in both young and aged mice.

**FIGURE 4:**
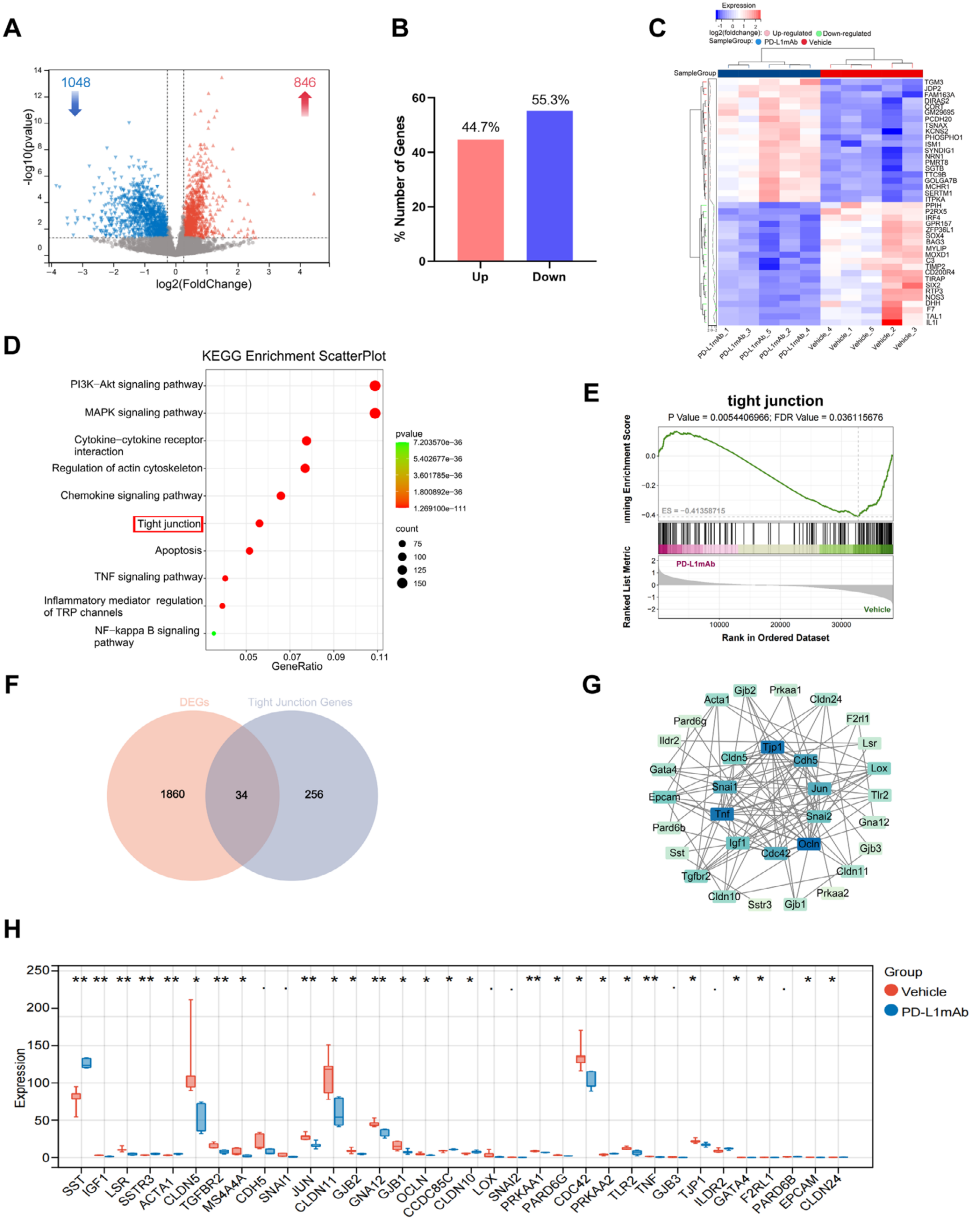
Transcriptomic profiling reveals PD‐L1 mAb‐induced gene expression changes and tight junction pathway enrichment in young ischemic mice. (A) Volcano plot visualizing the distribution of DEGs, highlighting 846 upregulated and 1048 downregulated genes. (B) Bar chart showing the proportional distribution of 44.7% upregulated and 55.3% downregulated DEGs between the two groups. (C) Heatmap illustrating the expression of the top 20 DEGs between the MCAO group and the PD‐L1 mAb treatment group (|FC| > 1.2, *p* < 0.05, *n* = 5). (D) KEGG pathway enrichment analysis of DEGs, presenting the top 20 significantly enriched pathways. (E) GSEA showing significant enrichment of the tight junction pathway. (F) Venn diagram identifying 34 key genes by intersecting DEGs with genes associated with tight junction pathways. (G) PPI network illustrating the relationships among the 34 tight junction‐related key genes, with color intensity representing protein importance within the network. (H) Box plot demonstrating the expression levels of the 34 tight junction‐related genes in the two groups (*p < 0.05, **p < 0.01).

After 3 days of reperfusion, PD‐L1 mAb treatment significantly reduced infarct volume in young mice compared to aged mice. Interestingly, our results showed that the infarct volume in aged mice was significantly smaller than in young mice (Figure 3C). The survival rates showed a trend toward improvement in the young PD‐L1 mAb‐treated group compared to aged mice (*p* = 0.0753) (Figure 3B). In contrast to the infarct volume results, behavioral deficits following ischemic stroke were more severe in aged mice than in young mice. However, neurological function recovery, assessed through mNSS, foot fault, and cylinder test, demonstrated significant improvement over time in young mice treated with PD‐L1 mAb compared to aged mice (Figure 4D). These results highlight the age‐dependent therapeutic benefits of PD‐L1 mAb in ischemic stroke recovery.

### 3.4 PD‐L1 mAb Modulates Immune and Tight Junction Signaling Pathways in Young Mice

Given the crucial role of PD‐L1 in immune suppression and its potential impact on brain injury, we first conducted RNA sequencing to investigate the effects of PD‐L1 mAb treatment on potential protective mechanisms in young mice. The results revealed that PD‐L1 mAb treatment caused the upregulation of 846 genes (44.7%) and the downregulation of 1048 genes (55.3%) compared to the vehicle group (|FC| > 1.2, *p* < 0.05), with downregulated genes being more predominant (Figure 4A,B). Figure 4C displays the top 20 upregulated and downregulated genes in the PD‐L1 mAb group compared to the vehicle group.

KEGG pathway enrichment analysis of these DEGs showed that the differentially expressed genes were associated with several pathways, including PI3K‐AKT signaling, MAPK signaling, cytokine–cytokine receptor interactions, tight junction proteins, and apoptosis (Figure 4D). Gene set enrichment analysis (GSEA) confirmed significant enrichment in the tight junction pathway (Figure 4E).

Further analysis identified 34 key genes involved in tight junction regulation by intersecting DEGs with a tight junction gene set (Figure 4F). The PPI network highlights the interrelationships of these 34 tight junction‐related genes, with nodes indicating their importance in the network (Figure 4G). These findings underscore the critical role of tight junction pathways in mediating the ischemic injury response and the therapeutic impact of PD‐L1 mAb.

### 3.5 PD‐L1 mAb Enhances Blood–Brain Barrier Integrity in an Age‐Dependent Manner

**FIGURE 5:**
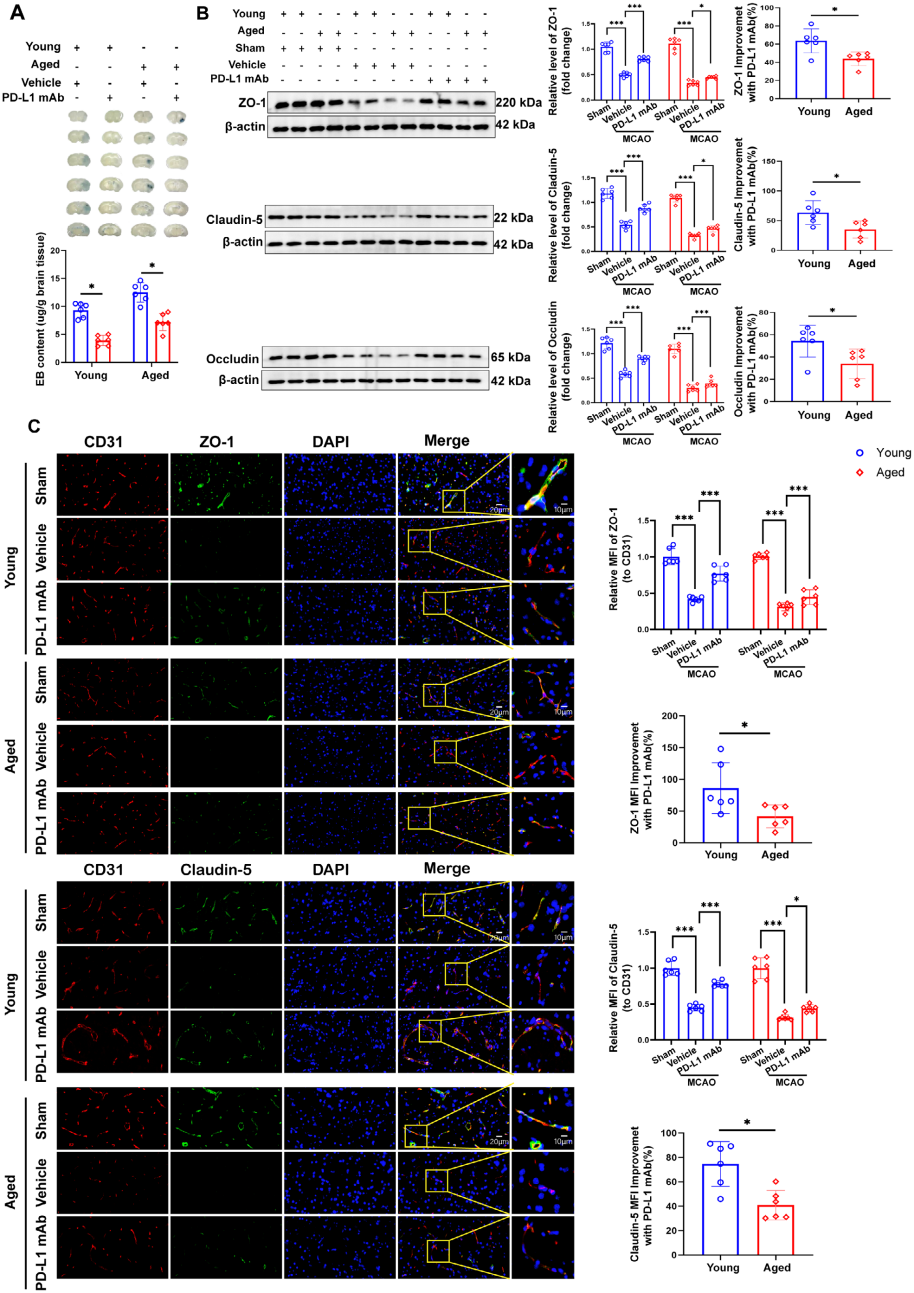
PD‐L1 mAb enhances BBB integrity more effectively in young mice than in aged mice following ischemic stroke. (A) Representative images of EB staining show reduced BBB permeability in PD‐L1 mAb‐treated groups compared to vehicle groups in both young and aged mice, with quantification indicating a greater effect in young mice. (B) Western blot analysis of ZO‐1, Claudin‐5, and Occludin levels in cortical tissue 72 h postischemia demonstrates significantly higher expression in PD‐L1 mAb‐treated groups, with quantification showing greater improvements in young mice. (C) Immunofluorescence costaining of CD31 (red) with ZO‐1 (green) or Claudin‐5 (green) in the peri‐infarct area highlights tight junction protein localization in CD31+ endothelial cells. The highlighted squares show magnified regions, emphasizing colocalization of ZO‐1 or Claudin‐5 with CD31, indicating improved BBB integrity in PD‐L1 mAb‐treated mice compared to vehicle‐treated mice. Scale bars: 20 μm (low magnification) and 10 μm (high magnification). Data are expressed as mean ± SD (*p < 0.05; ***p < 0.001).

After we had screened the effects on PD‐L1 mAb in young mice, we further investigated the age‐dependent effects of PD‐L1 mAb treatment; we explored its protective mechanisms, particularly its role in maintaining BBB integrity. EB extravasation experiments demonstrated that PD‐L1 mAb significantly improved BBB integrity following ischemic stroke, with a more pronounced reduction in BBB permeability in young mice compared to aged mice (Figure 5A).

Western blot analysis demonstrated upregulated expression of tight junction proteins (ZO‐1, Claudin‐5, and Occludin) in PD‐L1 mAb‐treated mice, with significantly greater increases observed in young mice (Figure 5B). Immunofluorescence staining further confirmed enhanced expression of ZO‐1 and Claudin‐5 localized to CD31+ endothelial cells in the ischemic penumbra of treated mice (Figure 5C). Quantitative analysis of relative MFI indicated a stronger therapeutic effect of PD‐L1 mAb on BBB‐associated tight junction proteins in young mice compared to aged mice.

### 3.6 PD‐L1 mAb Exerts Age‐Dependent Anti‐Inflammatory and Antiapoptotic Effects

**FIGURE 6:**
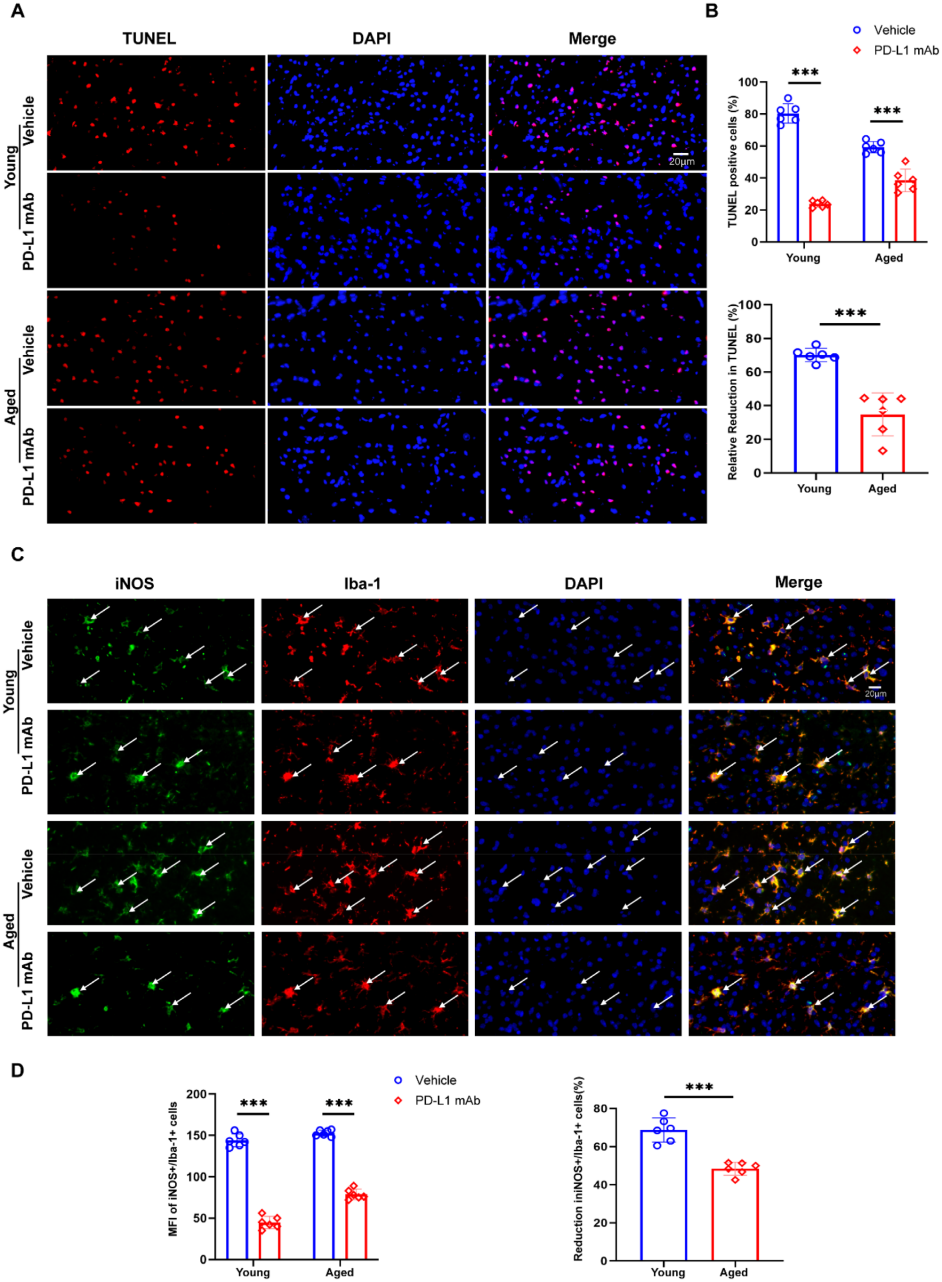
PD‐L1 mAb attenuates apoptosis and iNOS^+^/Iba1^+^ cells activation more effectively in young than aged mice following ischemic stroke. (A) TUNEL staining (red) with DAPI counterstain (blue) shows reduced apoptosis in PD‐L1 mAb‐treated groups for both young and aged mice. (B) Quantification indicates a significant reduction in TUNEL‐positive cells and a greater relative reduction in apoptosis in young mice compared to aged mice (*n* = 6). (C) Immunofluorescence staining for iNOS (green) and Iba1 (red) in the peri‐infarct area demonstrates a marked decrease in iNOS+/Iba1+ cells following PD‐L1 mAb treatment, with white arrows indicating colocalized cells. (D) Quantification shows a significant reduction in MFI of iNOS+/Iba1+ cells and a greater relative reduction in young mice compared to aged mice (*n* = 6). (***p < 0.001).

TUNEL staining revealed a greater reduction in apoptotic cells in the peri‐infarct area of young mice treated with PD‐L1 mAb compared to aged mice, further highlighting its age‐dependent efficacy (Figure 6A,B). Our group and previous studies have shown that peripheral immune cells infiltrate the brain after ischemic stroke, and Iba1+ cells also label infiltrating macrophages [22, 23]. Immunofluorescence staining of the peri‐infarct area demonstrated that PD‐L1 mAb significantly reduced the number of iNOS+/Iba1+ cells in young mice compared to aged mice, indicating a stronger anti‐inflammatory effect in younger animals (Figure 6C,D).

**FIGURE 7:**
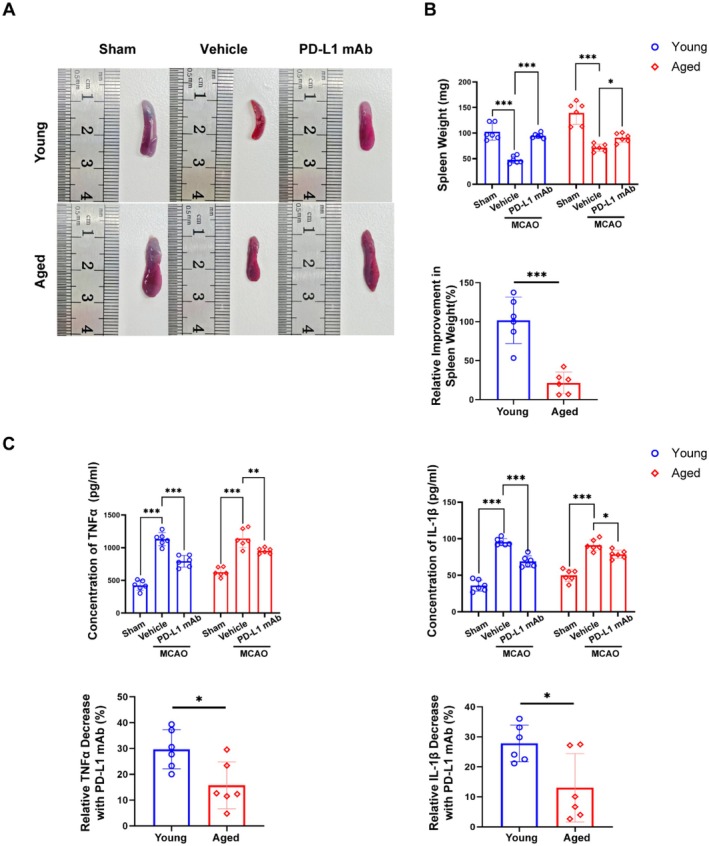
PD‐L1 mAb enhances splenic recovery and attenuates systemic inflammation more effectively in young than aged mice following ischemic stroke. (A) Representative images of spleens from Sham, Vehicle, and PD‐L1 mAb‐treated groups in young and aged mice. (B) Quantification of spleen weight shows a significant increase in PD‐L1 mAb‐treated groups compared to Vehicle groups, with a greater relative improvement in spleen weight in young mice (*n* = 6). (C) Pro‐inflammatory cytokine analysis reveals significant reductions in TNF‐α and IL‐1β levels in peripheral blood following PD‐L1 mAb treatment, with greater relative decreases observed in young mice compared to aged mice (*p < 0.05; **p < 0.01; ***p < 0.001).

Analysis of the splenic response showed that PD‐L1 mAb treatment enlarged spleens in both young and aged mice relative to their respective model groups, with a more pronounced effect observed in young mice (Figure 7A,B). Additionally, PD‐L1 mAb treatment significantly reduced peripheral levels of pro‐inflammatory cytokines, including TNF‐α and IL‐1β, with more pronounced effects in young mice than in aged mice (Figure 7C). These findings collectively highlight the enhanced therapeutic efficacy of PD‐L1 mAb treatment in young mice, particularly through modulation of multiple immune and inflammatory pathways.

## 4 Discussion

This study demonstrated a significant age‐dependent upregulation of PD‐L1, primarily in microglia and macrophages, as revealed by RNA sequencing and immunofluorescence. Young mice exhibited markedly higher PD‐L1 expression than aged mice, suggesting that immune adaptability plays a key role in ischemic stroke recovery. PD‐L1 mAb treatment significantly improved ischemic stroke outcomes, particularly in young mice, by reducing infarct volume, enhancing neurological recovery, and preserving BBB integrity through the upregulation of tight junction proteins, including ZO‐1, Claudin‐5, and Occludin. These protective effects were accompanied by a more pronounced reduction in neuroinflammation and apoptotic markers, and a more robust peripheral immune response. In aged mice, however, the therapeutic benefits were relatively modest, highlighting the influence of age‐dependent changes in immune regulation and vascular repair mechanisms.

Interestingly, consistent with previous studies [13, 14, 15, 16], our findings also support the observation that aged mice exhibit smaller infarct volumes than young mice despite experiencing more severe neurological deficits. This paradoxical finding has been reported in previous studies and may be attributed to several factors. One explanation is that aged mice often possess a more developed collateral circulation, which can limit infarct expansion but does not necessarily translate to better functional recovery [13]. Additionally, the metabolic demands of the aged brain are lower, potentially reducing acute ischemic damage [24]. However, despite a relatively smaller infarct size, aged mice exhibited worse behavioral outcomes, likely due to an exacerbated inflammatory response, persistent BBB disruption, and reduced neuroplasticity [13, 14]. The amplified neuroinflammatory cascade in aged mice, characterized by prolonged activation of pro‐inflammatory macrophages and increased neutrophil infiltration, may contribute to secondary neuronal injury, impairing functional recovery [13, 14, 25]. Furthermore, aging is associated with structural and functional impairments in neural circuits, limiting the ability to compensate for even minor tissue loss [11].

Given these observations, studying PD‐L1‐targeted therapies in young and aged ischemic stroke models is essential for understanding the broader implications of immune checkpoint modulation in ischemic stroke treatment. Current studies on PD‐L1 blockade have been conducted in young ischemic stroke models [5], creating a significant age gap in our knowledge regarding its therapeutic potential in the aging population. This discrepancy is particularly important because the majority of ischemic stroke patients are middle‐aged or elderly [26], making it imperative to assess whether immunomodulatory therapies like PD‐L1 mAb are equally effective across different age groups. Our findings suggest that while PD‐L1 blockade yields significant neuroprotection in young mice, its benefits in aged mice are attenuated, likely due to age‐related impairments in immune resolution and vascular repair. This highlights the necessity of age‐stratified therapeutic strategies, where different approaches may be required to optimize ischemic stroke treatment in younger versus older individuals.

Previous studies, such as those by Bodhankar et al., have shown that PD‐L1 signaling plays a key role in modulating both central and peripheral immune responses following ischemic stroke [5]. Our findings build upon this work by demonstrating that PD‐L1 expression and function are age‐dependent, which may explain the differential therapeutic response observed in young and aged mice. PD‐L1 signaling attenuates excessive activation of cytotoxic CD8+ T cells, thereby reducing secondary neuronal injury [5, 27], and facilitating macrophage polarization toward an anti‐inflammatory phenotype [27]. This immunoregulatory effect appears to be more pronounced in young mice, aligning with their greater neuroplasticity and more efficient vascular repair mechanisms [28, 29].

Transcriptomic analysis revealed enrichment of both apoptosis and tight junction pathways in PD‐L1 mAb‐treated young mice. However, such enrichment reflects gene set overrepresentation rather than functional activation. Notably, despite apoptosis pathway enrichment, TUNEL staining showed reduced neuronal apoptosis suggesting nontranscriptional mechanisms or indirect effects via immune suppression and PI3K/Akt signaling [30, 31]. Likewise, the enrichment of tight junction‐related genes corresponded with the observed upregulation of ZO‐1, Claudin‐5, and Occludin, supporting enhanced BBB integrity. Together, these results indicate that PD‐L1 mAb confers neuroprotection by modulating structural and immune‐regulatory pathways.

The diminished response to PD‐L1 mAb in aged mice may be attributed to multiple factors. First, aged mice exhibited lower baseline PD‐L1 expression, suggesting a reduced capacity for immune modulation following ischemic injury [7, 32, 33]. This aligns with the concept of “inflamm‐aging,” a state of chronic low‐grade inflammation that is chronic low‐grade inflammation associated with impaired immune resolution and prolonged activation of inflammatory pathways [34, 35]. Second, BBB disruption was more severe in aged mice, potentially limiting the ability of PD‐L1 blockade to preserve vascular integrity [36, 37]. Third, metabolic differences between young and aged brains may also contribute to variations in ischemic vulnerability, with older brains exhibiting lower baseline metabolic activity and distinct proteomic adaptations that influence cell survival and recovery [13].

Despite these challenges, our findings highlight the necessity of investigating PD‐L1‐targeted therapies across different age groups. Given that the majority of ischemic stroke patients are middle‐aged or elderly [26], understanding how aging alters immune responses and treatment efficacy is crucial for clinical translation. While PD‐L1 mAb therapy may be more effective in younger individuals, it remains to be determined whether combinatorial strategies, such as adjunctive therapies targeting immune senescence or vascular dysfunction, could enhance its efficacy in older patients.

Although PD‐L1 mAb demonstrated significant neuroprotective effects in our study, we did not evaluate its direct impact on cerebral perfusion. Laser speckle imaging was used solely to confirm the success of MCAO modeling before treatment, and no posttreatment assessment of blood flow was conducted. Therefore, we cannot determine whether PD‐L1 mAb influences cerebral blood flow. However, since the antibody was administered after the reperfusion phase and current literature does not support a direct vascular effect of PD‐L1 blockade, we cautiously speculate that the observed therapeutic benefits are more likely attributable to immune modulation rather than changes in cerebral perfusion. Future studies designed to investigate vascular responses to PD‐L1‐targeted therapies will be necessary to address this question.

This study has several limitations. First, it focuses on the acute phase of ischemic stroke (within 3 days following ischemic stroke) and does not evaluate long‐term outcomes such as functional recovery, inflammation resolution, or neurovascular remodeling. Second, all experiments were performed in male mice; considering well‐established sex differences in ischemic stroke pathology and immune responses, the lack of female models may introduce a sex‐related bias and limit generalizability. Third, although this study reveals key mechanisms by which PD‐L1 contributes to immune modulation and neuroprotection, most molecular and transcriptomic analyses were conducted in young mice. The limited mechanistic data from aged mice restrict a full understanding of age‐dependent therapeutic effects. Lastly, the translational relevance of PD‐L1‐targeted therapy in human ischemic stroke remains to be validated, especially in the presence of age‐related comorbidities that may influence treatment efficacy.

## 5 Conclusion

In conclusion, our study provides new insights into the age‐dependent role of PD‐L1 in immune modulation and vascular repair following ischemic stroke. The findings underscore the importance of considering age as a critical factor in immunotherapeutic strategies for ischemic stroke and support the need for further investigations to optimize treatment approaches for different patient populations.

## Author Contributions

Hang Hang and Can Xu contributed equally to this work and share first authorship. Hang Hang was responsible for the overall experimental design, data analysis, figure preparation, and drafting of the manuscript. Can Xu was primarily responsible for the establishment of the animal model and the intervention experiments. Likun Wang, Cuiying Liu, and Rongping Zhang provided essential support and guidance on experimental design, platform operation, and data acquisition. Guofeng Wu and Heng Zhao conceived the study, supervised the data interpretation, and led the manuscript revision and final approval. All authors reviewed the manuscript.

## Disclosure

The authors have nothing to report.

## Ethics Statement

This study has been approved by the Capital Medical University Animal Ethics Committee.

## Conflicts of Interest

The authors declare no conflicts of interest.

## Supporting information


Data S1.

## Data Availability

The data that support the findings of this study are available from the corresponding author upon reasonable request.
