# Supplementary figures and images for: Targeting PD‐L1 for Ischemic Stroke Recovery: Age‐Dependent Modulation of Immune and BBB Pathways
Source: CNS Neurosci Ther. 2025 Jul 23;31(7):e70523. doi: 10.1111/cns.70523 (PMC12287378; doi:10.1111/cns.70523)

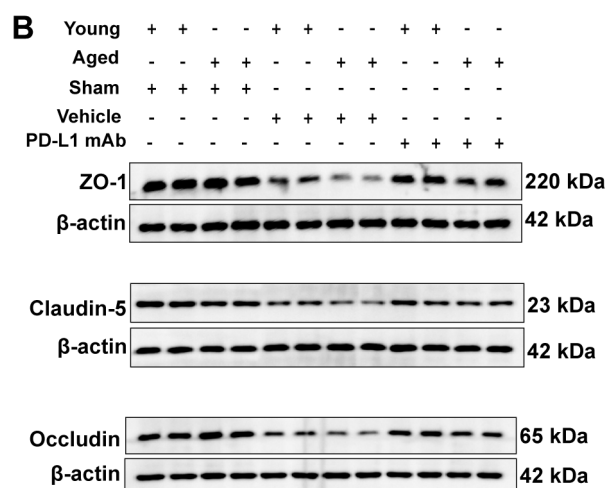

A. ZO-1 (220kDa)

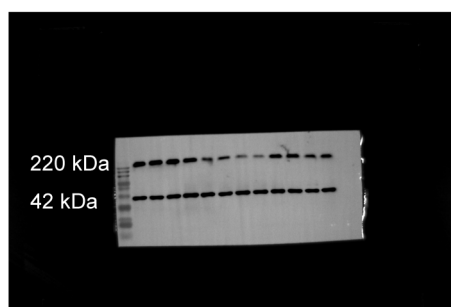

B. Claudin-5 (23kDa)

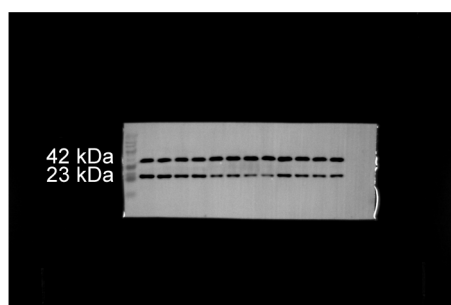

C. Occludin (65kDa)

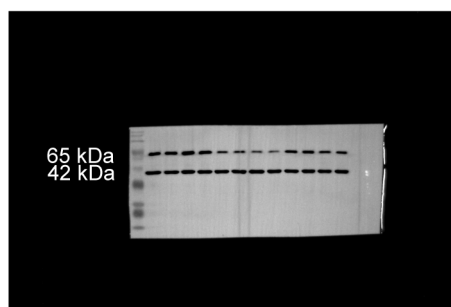

Supplement: Supplementary file 1 — Data S1. [file CNS-31-e70523-s001.pdf]
